# HMPV Impairs Macrophage Phagocytosis Through a Replication-Dependent Mechanism Associated with Reduced CD36 Expression and the Viral G Protein

**DOI:** 10.3390/v18060649

**Published:** 2026-06-04

**Authors:** Iván Martínez-Espinoza, Pius I. Babawale, Basel Abuaita, Antonieta Guerrero-Plata

**Affiliations:** Department of Pathobiological Sciences, School of Veterinary Medicine, Louisiana State University, Baton Rouge, LA 70803, USA

**Keywords:** HMPV, human metapneumovirus, phagocytosis, CD36, virus, respiratory

## Abstract

Human metapneumovirus (HMPV) is a major cause of respiratory infections, but its impact on macrophage antibacterial functions remains poorly understood. Macrophages play a crucial role in host defense through phagocytosis, and impairment of this function may increase susceptibility to secondary infections. Here, we show that HMPV infection of THP-1-derived macrophages significantly reduces bacterial uptake in a replication-dependent manner. This effect was restricted to infected cells and was not recapitulated by cell-free supernatants, indicating a cell-intrinsic mechanism. HMPV infection was also associated with reduced expression of the scavenger receptor CD36. Viral gene knockdown studies further implicated the HMPV G protein in this phenotype, as silencing the G protein restored phagocytic function. Analysis of single-cell RNA-sequencing datasets from HMPV-infected mouse lungs revealed reduced CD36 expression and broader alterations in phagocytosis-associated gene programs across lung macrophage subsets. Supporting these observations, expression of *Cd36* and *Marco* was reduced in lung tissue from HMPV-infected mice. Overall, these findings demonstrate that HMPV impairs macrophage-mediated bacterial uptake through a replication-dependent, cell-intrinsic mechanism and identify reduced scavenger receptor expression and the viral G protein as factors associated with this phenotype. These alterations may contribute to increase susceptibility to secondary bacterial infections during HMPV infection.

## 1. Introduction

Human metapneumovirus (HMPV) is a leading cause of acute respiratory tract infections worldwide, particularly affecting young children, older adults, and immunocompromised individuals [1,2,3,4,5,6,7,8]. Since its initial isolation in the Netherlands in 2001, HMPV has been increasingly recognized for its clinical significance in both community and nosocomial settings [9,10,11]. The virus is primarily associated with upper and lower respiratory tract diseases, ranging from mild cold-like illnesses to severe bronchiolitis and pneumonia [12]. Epidemiologic studies indicate that HMPV accounts for approximately 5–15% of acute respiratory tract infections in young children annually, with activity peaking in late winter and spring [6,13,14,15,16,17]. In the United States, HMPV is estimated to cause nearly one million outpatient visits and over 260,000 emergency department visits annually among children under five, reflecting its considerable contribution to healthcare utilization [10,18]. Serological data demonstrate that up to 80–90% of children are infected by five years of age, underscoring its near-universal prevalence in early childhood [1,2,4,6,13,15,18,19,20,21]. In addition to its direct pathogenic effects, HMPV infection can also modulate hosts’ immune responses, including those of innate immune cells such as macrophages [22,23].

Macrophages are pivotal in maintaining pulmonary homeostasis and orchestrating early immune defense against invading pathogens [23]. During HMPV infection, macrophages are both activated and modulated by the virus, inducing innate immune programs, such as interferon and proinflammatory cytokines, that can differ from those triggered by related respiratory viruses [24,25]. These responses are mediated by specific innate receptors and signaling pathways [26,27]. Furthermore, HMPV has been shown to alter macrophage antibacterial functions by dampening IL-1β induction in response to bacterial stimuli [28]. In vivo depletion studies also indicate that alveolar macrophages contribute to HMPV-associated lung pathology [29].

Secondary bacterial infections often complicate viral respiratory illnesses by exploiting immune dysregulation induced by the viral infection. In adult cohorts with HMPV lower respiratory infection, *Staphylococcus aureus* (*S. aureus*) and other bacteria have been co-detected [30,31]. This repeated co-detection suggests that HMPV infection can predispose to bacterial colonization or superinfections through multiple mechanisms, including phagocytosis, by which internalized bacteria are destroyed [32,33]. Compromise of macrophage function can therefore have profound consequences for the outcome of respiratory infections [34]. However, whether HMPV alters macrophage phagocytic capacity and the mechanisms by which this may impair bacterial clearance remain unknown.

In this study, we show that HMPV infection impairs the phagocytic capacity of THP-1-derived macrophages in a replication-dependent manner. This phenotype is partially influenced by the viral attachment protein G and is associated with reduced CD36 expression, which we further validated in monocyte-derived macrophages (MDMs). Publicly available scRNA-seq datasets also supported a broader reduction in phagocytosis-related programs, including *Cd36* and *Marco*, across pulmonary macrophage subsets, a pattern further confirmed by RT-qPCR analysis of lung tissue from infected mice. Together, these findings suggest that HMPV impairs macrophage-mediated bacterial uptake by altering key receptors involved in phagocytosis.

## 2. Materials and Methods

### 2.1. Cell Culture

All cell lines were obtained from the American Type Culture Collection (ATCC). The human monocytic THP-1 cell line (ATCC, TIB-202, Manassas, VA, USA) was cultured in RPMI 1640 (HyClone, Logan, UT, USA) supplemented with 10% fetal bovine serum (FBS) (Biowest, Riverside, MO, USA) and 1% penicillin–streptomycin (Gibco, Gaithersburg, MD, USA), herein referred to as complete medium. Cells were incubated with 5% CO_2_ at 37 °C. THP-1 monocyte differentiation into macrophages was performed in the presence of Phorbol 12-myristate 13-acetate (PMA) (Millipore Sigma, Burlington, MA, USA) as previously reported [24]. Peripheral blood mononuclear cells (PBMCs) were obtained from de-identified buffy coat preparations provided by the Our Lady of the Lake Blood Donor Center. As investigators had no access to donor-identifying information, these specimens were not considered human subjects research under HHS regulations, 45 CFR Part 46 [35]. Accordingly, Institutional Review Board review and written informed consent were not required. PBMCs were differentiated into monocyte-derived macrophages (MDMs) for 7 days in the presence of 100 ng/mL of granulocyte-macrophage colony-stimulating factor (GM-CSF) (Peprotech, Cranbury, NJ, USA), as previously reported [25].

### 2.2. Virus Stocks

HMPV strain CAN97-83 and HMPV-GFP (HMPV CAN97-83 expressing the green fluorescent protein) were obtained from the Centers for Disease Control and Prevention (CDC, Atlanta, GA, USA), and ViraTree (LLC, Research Triangle Park, NC, USA), respectively. The viruses were propagated in LLC-MK2 cells in MEM containing 1 μg trypsin/mL (Worthington Biochemicals, Lakewood, NJ, USA) and sucrose purified as previously reported [36,37]. HMPV viral titers (PFU/mL or FFU/mL) were quantified in LLC-MK2 cells using a combined methylcellulose plaque assay and cell-based immunoassay, as previously described [38].

### 2.3. Viral Infection

THP-1-macrophages were infected with HMPV at a multiplicity of infection (MOI) of 1.0 in the presence of 1 μg/mL of trypsin (Worthington Biochemical, Lakewood, NJ, USA) in plain RPMI 1640 medium (Cytiva, Marlborough, MA, USA). After 2 h of viral adsorption, the inoculum was removed, and 1 mL of complete RPMI medium was added to each well.

### 2.4. Bacteria Propagation

*S. aureus* USA300 JE2 strain was streaked on tryptic soy agar (TSA; BD, Franklin Lakes, NJ, USA), and a single colony was selected and grown overnight at 37 °C with shaking (200 rpm) in tryptic soy broth (TSB) (BD, Franklin Lakes, NJ, USA). For infections, bacterial cells were pelleted, washed with phosphate-buffered saline (PBS), and resuspended in PBS. The inoculum was estimated by measuring the optical density at 600 nm (OD_600_).

### 2.5. Phagocytosis Assay

We evaluated the phagocytic capacity of HMPV-infected macrophages using fluorescently labeled bacteria. *S. aureus* was used in these experiments as a defined phagocytic target using the IncuCyte^®^ live-cell analysis (Sartorius, Bohemia, NY, USA). This method provided a dynamic and quantitative evaluation of bacterial uptake and processing within macrophages. The bacteria were labeled, according to the manufacturer’s instructions, with pHrodo™ (Thermo Fisher Scientific, Waltham, MA, USA), a pH-sensitive fluorogenic substrate that fluoresces upon acidification in the phagolysosome, allowing the quantification of actively phagocytosed bacteria [39]. Labeled bacteria were then resuspended in PBS for the phagocytosis assay. Macrophages were first infected with HMPV at an MOI of 1.0. This MOI was chosen because it achieved robust infection with minimal cytotoxicity, as previously reported [24]. After 48 h of infection, they were detached, counted, and seeded (2.5 × 10^5^ cells/well) in a 24-well plate. After attachment, cells were then incubated with pHrodo-labeled *S. aureus* at an MOI of 40 for 2 h. Phagocytosis was quantified using the Incucyte^®^ Zoom HD/2CLR time-lapse microscopy system (Sartorius, Bohemia, NY, USA). Fluorescence intensity was measured, normalized to uninfected controls, and expressed as a percentage of phagocytosis.

### 2.6. Bacteria Killing Assay

For the *S. aureus* killing assay, macrophages were infected with HMPV and seeded in 6-well plates, as indicated above, at an MOI of 1. Forty-eight hours after infection, the cells were detached, counted, and seeded at a density of 2.5 × 10^5^ cells/well in antibiotic-free complete medium. After allowing them to adhere, macrophages were challenged with *S. aureus* at an MOI of 40 for 2 h, followed by three washes with PBS and then incubated in RPMI medium containing 100 µg/mL gentamicin (Gibco, Gaithersburg, MD, USA) to kill extracellular bacteria. To enumerate intracellular bacteria, macrophages were washed twice with PBS, lysed with 0.1% NP-40 (Thermo Fisher Scientific, Waltham, MA, USA), and the lysates were plated on tryptic soy agar for 24 h. Colonies were counted, and the concentration of bacteria as colony-forming unit per ml (CFU/mL) was calculated.

### 2.7. Flow Cytometry

Macrophage phagocytosis and CD36 expression were also evaluated by flow cytometry. For CD36 expression, macrophages were infected with HMPV, and 48 h after infection, cells were detached and washed with PBS containing 0.5% BSA (PBS/BSA). Fc receptors were then blocked using FcR blocking reagent (Miltenyi Biotec, Gaithersburg, MD, USA). Next, the cells were stained with an APC-conjugated anti-human CD36 antibody (eBioscience, San Diego, CA, USA) or an isotype control (mouse IgG1κ, eBioscience, San Diego, CA, USA), then fixed with 1% formaldehyde. For the phagocytosis assay, macrophages were exposed to pHrodo™-labeled *S. aureus* for 2 h, then detached and washed with PBS/BSA. Cells were fixed with 1% formaldehyde. Samples stained for detection of CD36 or pHrodo were acquired on a Fortesa or FACScan flow cytometer, respectively (BD Biosciences, Franklin Lakes, NJ, USA). Data were analyzed with FlowJo v10.10.0 software (BD Biosciences, Franklin Lakes, NJ, USA).

### 2.8. ROS Measurement

To quantify reactive oxygen species (ROS) production, both uninfected and infected macrophages were labeled with the fluorescent probe CM-H_2_DCFDA (Thermo Fisher Scientific, Waltham, MA, USA). Macrophages infected with HMPV for 24 or 48 h were detached, washed, and incubated with CM-H_2_DCFDA (10 µM) in serum-free medium for 30 min at 37 °C in the dark, allowing the probe to load into the cells. Unincorporated dye was then removed by washing with PBS/BSA. Samples were further analyzed by flow cytometry.

### 2.9. Gene Expression and Viral RNA Quantification by qRT-PCR

Total RNA was extracted from cell lysates to quantify viral genome copies and CD36 transcript levels using the RNeasy Plus kit (Qiagen, Germantown, MD, USA) following the manufacturer’s protocol. Complementary DNA (cDNA) was synthesized from purified RNA using qScript cDNA SuperMix (QuantaBio, Beverly, MA, USA). Quantitative PCR amplification was carried out with PowerTrack SYBR Green Master Mix (Thermo Fisher Scientific, Waltham, MA, USA) on a QuantStudio™ 12K Flex system (Applied Biosystems, Foster City, CA, USA). Predesigned primers targeting human *CD36*, HMPV-G, HMPV-M2-2, HMPV-SH, HMPV-N, mouse *Cd36* and *Marco*, and both human and mouse *GAPDH* (Integrated DNA Technologies, Coralville, IA, USA) were used. Relative transcript levels were determined using the ΔΔCt method with GAPDH as the housekeeping control. Data acquisition and analysis were performed using QuantStudio™ 12K Flex Software v1.3.

### 2.10. Generation of CRISPR/Cas9 THP-1 Gene Knockout and Stable THP-1 Cell Lines Expressing shRNAs

CD36 knockout (CD36 KO) THP-1 cells were generated using the LentiCRISPR v2 single-vector system (Addgene, Watertown, MA, USA). Single guide RNA (sgRNA) sequences targeting CD36 were designed and cloned into LentiCRISPR v2 at the BsmBI restriction sites. The resulting lentiviral construct was then co-transfected with psPAX and pVSV-G packaging plasmids (Addgene, Watertown, MA, USA) into 293FT cells using Lipofectamine™ 3000 (Invitrogen, Waltham, MA, USA). After 48 h, the supernatant containing lentiviral particles was harvested and used to transduce THP-1 cells in the presence of 10 µg/mL Polybrene (Millipore Sigma, Hayward, CA, USA). Successful transductants were selected with 10 µg/mL Puromycin (Gibco, Gaithersburg, MD, USA). To obtain monoclonal cell lines, these puromycin-resistant cells were seeded at limiting dilution, allowing individual clones to be isolated. Each clone was then expanded and validated for CD36 KO using RT-qPCR, Capillary Western blot, and flow cytometry to confirm loss of gene, intracellular, and surface CD36 expression, respectively. Stable cell lines constitutively expressing shRNAs against HMPV G and M2 genes were generated using the pLKO-TCR lentiviral vector (Addgene, Watertown, MA, USA). Short hairpin RNA (shRNA) sequences specific to each HMPV gene were designed and inserted into pLKO-TCR via EcoRI and AgeI restriction sites. For lentivirus production, 293FT cells were co-transfected with the shRNA-containing pLKO-TCR construct, psPAX (packaging plasmid), and pVSV-G (envelope plasmid) using Lipofectamine™ 3000 (Invitrogen, Waltham, MA, USA). After 48 h, the supernatants containing lentiviral particles were collected and used to transduce early passage THP-1 cells. Polybrene (Millipore Sigma, Hayward, CA, USA) was added to a final concentration of 10 µg/mL to enhance transduction efficiency. Following transduction, the cells were selected with 10 µg/mL puromycin (Gibco, Gaithersburg, MD, USA). Surviving clones were expanded and validated to confirm the knockdown of each target HMPV gene.

### 2.11. Capillary Western Blot Analysis Using the ProteinSimple Wes System

Protein expression levels were evaluated by a capillary-based immunoassay on the ProteinSimple Wes System (ProteinSimple, Santa Clara, CA, USA). This method allowed quantitative protein detection with high sensitivity and reproducibility, while simultaneously normalizing expression to housekeeping proteins for accurate comparison between conditions. Whole-lysate extracts were initially diluted in 0.1× Sample Buffer, after which four parts of the diluted samples were combined with one part of 5× Fluorescent Master Mix (containing 5× sample buffer, 5× fluorescent standard, and 200 mM DTT). This mixture was heated to 95 °C for 5 min to achieve protein denaturation. Following denaturation, the prepared samples, blocking reagent, primary antibodies (diluted 1:10 for CD36 (Santa Cruz Biotechnology, Dallas, TX, USA), 1:100 for HMPV, and 1:1000 for GAPDH (Cell Signaling Technology, Danvers, MA, USA)), HRP-conjugated secondary antibodies, and the chemiluminescent substrate were aliquoted into designated wells of an assay plate as indicated in the standardized protocol. A biotinylated ladder was used as a molecular weight marker in each run. Once the plate was loaded, the instrument automatically performed both electrophoretic separation and immunodetection in each capillary, thereby streamlining the entire analysis process. All signal detection and analysis were conducted using Compass for Simple Western software (ProteinSimple, Santa Clara, CA, USA). The software calculates the area under the curve (AUC) for each detected peak, which was then used to quantify the relative abundance of the target proteins across experimental conditions.

### 2.12. Single-Cell RNA Sequencing (scRNA-Seq) Analysis

Publicly available scRNA-seq data were retrieved from the Gene Expression Omnibus (GEO) database (accession number: GSE261511), originally generated by Sojati et al. [40], and processed locally in R (v4.2.2) using Seurat (v4.3.0). Raw 10X Genomics count matrices were downloaded from GEO, imported, and merged with sample-level metadata (mock vs. HMPV; biological replicates). Cells underwent standard quality-control filtering based on detected genes, total UMI counts, and mitochondrial transcript percentage, followed by normalization and variance stabilization (SCTransform), dimensionality reduction (PCA), UMAP embedding, and unsupervised graph-based clustering. Macrophages were identified and enriched based on robust expression of canonical myeloid/macrophage markers *Adgre1* (*F4/80*), *Csf1r*, *Lyz2*, *Cd68* (with supportive myeloid genes such as *Tyrobp*), together with lineage-exclusion of non-myeloid populations by screening for epithelial (*Krt8*, *Krt18*, *Epcam*), endothelial (*Pecam1*, *Kdr*, *Vw*f), and lymphoid/NK markers (*Cd3d*, *Cd3e*, *Trac*, *Ms4a1*, *Cd79a*, *Nkg7*). After removing non-macrophage-like cells, the retained macrophage compartment was re-normalized, re-embedded (PCA/UMAP), and reclustered to refine within-lineage structure. Macrophage subpopulations were annotated using established subtype signatures—alveolar macrophages by *Siglecf*, *Pparg*, *Car4*; interstitial macrophages by *Cx3cr1*, *Cd74*, *H2-Ab1*; and inflammatory/monocyte-derived macrophages by *Ly6c2*, *Ccr2*, *Il1b*, *S100a8/S100a9*—assigning identities based on relative enrichment of these gene sets across clusters. To evaluate in vivo relevance, we quantified and visualized expression of curated phagocytosis-associated receptors—including *Cd36* and other scavenger, Fc, complement, and lectin receptors—across macrophage subsets and experimental conditions. Figures and plots were generated using ggplot2 (v3.4.x) and base R. mice.

### 2.13. In Vivo Validation of Cd36 and Marco Gene Expression

Female 8–10-week-old BALBc mice were purchase from Envigo (Indianapolis, IN, USA). Mice were infected intranasally with HMPV diluted in sterile PBS at a final dose of 1 × 10^7^ PFUs in a total volume of 50 μl. Mock-infected animals received 50 μl sterile PBS. Lung tissues were collected at day 1 p.i. for RNA extraction and analysis of Cd36 and Marco gene expression. All animals were housed in specific pathogen-free conditions in accordance with the Louisiana State University Institutional Animal Care and Use Committee (protocol No. 15-062). No additional animal experiments were performed in this study. RNA extraction, cDNA synthesis, and qRT-PCR were performed as described above. Relative gene expression was calculated using the ΔΔCt method with GAPDH as the housekeeping control.

### 2.14. Statistical Analysis

Statistical analyses were performed using unpaired Student’s *t*-tests and one-way ANOVA to compare differences among experimental groups, followed by appropriate post hoc tests to correct for multiple comparisons. All analyses were conducted with GraphPad Prism v10.6.0 (GraphPad Software, Boston, MA, USA).

For the scRNA-seq validation analysis, we performed it in R (v4.5.2) using Seurat (v5.4.0) and SeuratObject (v5.3.0) for preprocessing and cell annotation, and edgeR with limma-voom (limma) for pseudobulk differential expression. To avoid cell-level pseudoreplication, raw RNA counts were aggregated at the biological sample level within each macrophage subtype, normalized by TMM, and filtered to retain genes with CPM > 1 in at least two samples before modeling. Differential expression between HMPV and mock conditions was tested using voom-transformed counts fit to linear models with empirical Bayes moderation, and multiple testing correction was performed using the Benjamini–Hochberg procedure; therefore, adjusted *p* values (adj.P.Val) are reported as false discovery rates (FDR), with FDR < 0.05 considered significant. Per-cell visualizations (UMAPs and boxplots) were generated for descriptive purposes, and any significance annotations shown on these plots reflect subtype-specific pseudobulk FDR results rather than cell-level statistical tests.

## 3. Results

### 3.1. HMPV Infection Decreases the Phagocytic Capacity of Macrophages

Macrophages play a crucial role in pathogen clearance, and their ability to engulf and eliminate bacteria is essential for host defense. HMPV infection in children has been associated with an increased risk of secondary bacterial infections [3,5,30,31,41], suggesting that viral infection may compromise macrophage function.

**Figure 1:**
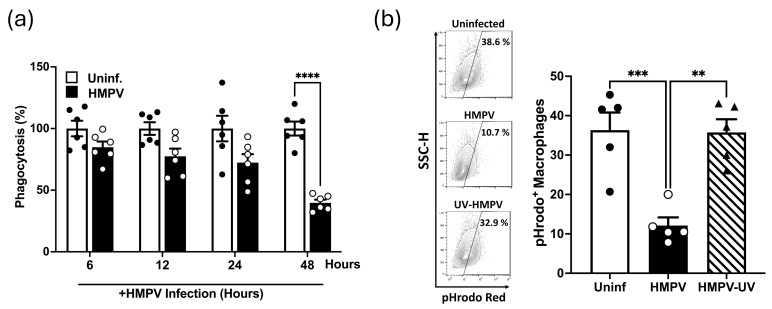
Phagocytic capacity of macrophages exposed to HMPV infection. Macrophages were infected with HMPV at an MOI of 1.0, and phagocytosis was assessed at 6, 12, 24, and 48 h post-infection using pHrodo-labeled *S. aureus* (MOI 40). (**a**) Phagocytosis was quantified by Incucyte^®^ live-cell imaging system at each time point. Macrophages infected with either active HMPV or UV-inactivated HMPV were exposed to pHrodo-labeled *S. aureus. * pHrodo-positive cells were quantified by flow cytometry. (**b**) Representative dot plots and the corresponding quantification show the percentage of phagocytic macrophages. Data presented mean ± SEM (*n* = 6) from the indicated independent experiments. Statistical analysis was performed using (**a**) multiple unpaired *t*-tests or (**b**) ANOVA with Tukey’s post hoc test. ** *p* < 0.01, *** *p* < 0.001, **** *p* < 0.0001.

HMPV-infected macrophages were detached, counted, and re-seeded prior to incubation with pHrodo-labeled *S. aureus* for 2 h to control for potential cell death induced by infection and ensure that phagocytosis measurements were performed using viable macrophages. Phagocytosis was then assessed at 6, 12, 24, and 48 h post-HMPV infection using the IncuCyte^®^ live-cell imaging assay. Between 6 and 24 h post-infection, phagocytosis was only marginally reduced. However, by 48 h post-infection, macrophages showed a significant impairment of the phagocytic function, with approximately a 60% reduction compared with uninfected cells (Figure 1a). These results suggest that while macrophages initially maintain their ability to engulf bacteria, prolonged HMPV infection substantially compromises their phagocytic capacity. To further determine whether the observed reduction in phagocytosis was dependent on viral replication, macrophages were exposed to HMPV or UV-inactivated HMPV (HMPV-UV) for 48 h, followed by the assessment of phagocytosis by flow cytometry. This approach provides a precise, single-cell assessment, allowing accurate quantification of the percentage of bacteria-positive macrophages. As shown in Figure 1b, 36.2% of uninfected macrophages were bacteria-positive, whereas in HMPV-infected cells, this percentage dropped to 12.1%, representing a 64.6% reduction in phagocytosis and confirming significant impairment induced by HMPV. Notably, exposure to HMPV-UV did not impair phagocytic function, with 35.74% of cells showing phagocytosis of bacteria, comparable to uninfected controls. These data are consistent across both fluorescence imaging and flow cytometry, demonstrating that HMPV actively disrupts macrophage phagocytic capacity in a viral replication-dependent manner.

### 3.2. Direct Infection of Macrophages by HMPV Is Required to Impair Phagocytosis

**Figure 2:**
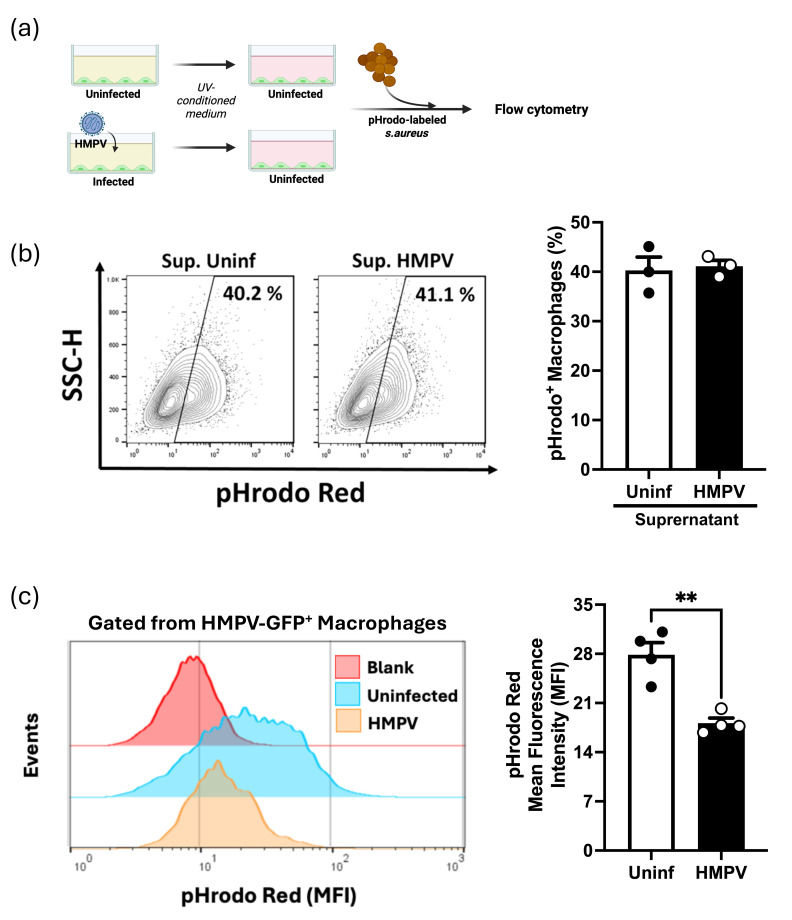
Intrinsic effect of HMPV on macrophage phagocytic function. (**a**) Supernatants from uninfected or HMPV-infected macrophages were transferred to uninfected macrophages for 24 h before the phagocytosis assay. Cells treated with supernatants from uninfected (Sup. Uninf) or HMPV-infected (Sup. HMPV) macrophages were then exposed to pHrodo-labeled *S. aureus* (MOI 40) for 2 h. (**b**) Representative flow cytometry plots (**left**) and quantification of pHrodo-positive macrophages (**right**) are shown (*n* = 3). (**c**) Macrophages infected with HMPV-GFP for 48 h were exposed to pHrodo-labeled *S. aureus* (MOI 40) for 2 h. Representative histograms gated on GFP-positive and GFP-negative macrophages are shown (**left**), with quantification of pHrodo red mean fluorescence intensity (MFI) on the right (*n* = 4). Data are presented as mean ± SEM from the indicated independent experiments. Statistical significance was determined using Student’s *t*-test. ** *p* < 0.01.

Cytokines produced during viral infections can impair macrophage phagocytic capacity, potentially contributing to immune dysfunction [42]. To determine whether a soluble factor mediated the phagocytic impairment observed in HMPV-infected macrophages, we collected supernatants from infected macrophages at 48 h post-infection and transferred them to uninfected macrophages for an additional 24 h (Figure 2a). These cells were subsequently challenged with pHrodo-labeled bacteria at an MOI of 40 for 2 h, and their phagocytic activity was assessed. As shown in Figure 2b, no difference in phagocytosis was observed between macrophages treated with supernatants from either infected or uninfected cells. Phagocytic percentages remained comparable, indicating that secreted factors are unlikely to account for the observed impairment in bacterial uptake.

To further assess whether the reduced phagocytic capacity was intrinsically linked to viral infection, macrophages were infected with HMPV-GFP. This strategy enabled the direct identification and analysis of infected (GFP-positive) macrophages and comparison of their bacterial uptake with that of uninfected (GFP-negative) cells. Macrophages were infected with HMPV-GFP at an MOI of 1.0 and, at 48 h post-infection, challenged with pHrodo-labeled red bacteria at an MOI of 40 for 2 h. Cells were detached, washed with PBS/BSA, and analyzed by flow cytometry to quantitatively assess phagocytosis. GFP-positive and GFP-negative macrophage populations were gated separately, allowing for HMPV-infected vs. uninfected groups comparisons between HMPV-infected and uninfected groups. For each macrophage group, the MFI of bacteria-positive cells was calculated and compared. As shown in Figure 2c, HMPV-infected macrophages (GFP-positive) exhibited a reduction in pHrodo MFI from 27.9 ± 1.7 to 18.1 ± 0.7, indicating that HMPV infection intrinsically impairs bacterial uptake at the cellular level.

### 3.3. HMPV Infection Does Not Compromise Macrophage Antibacterial Activity

Phagocytosis is a highly complex and regulated process that involves not only the initial recognition and internalization of a pathogen, but also its subsequent processing and degradation within the phagolysosome [32,33,43]. Once internalized, bacteria are subjected to oxidative and enzymatic degradation, ultimately leading to their clearance [43]. However, our assessment of phagocytosis relied on pHrodo-labeled fixed bacteria, which selectively detect bacteria that had reached the phagolysosome, thereby limiting our ability to determine whether the observed reduction in phagocytosis resulted from defective bacterial uptake or impaired intracellular degradation. To address this limitation, we sought to determine whether HMPV infection interferes with lysosomal degradation or bacterial internalization.

**Figure 3:**
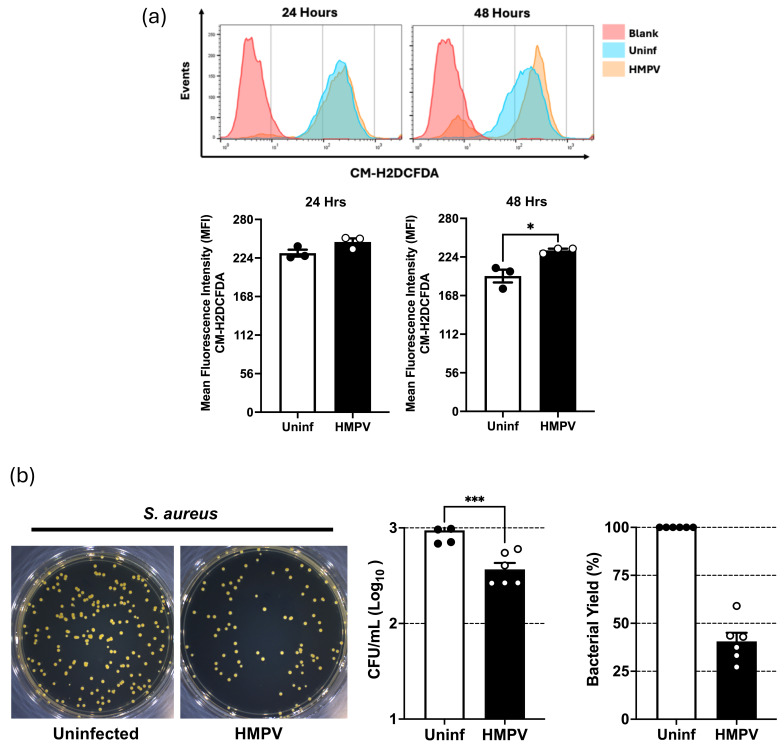
Antimicrobial activity in HMPV-infected macrophages. Macrophages were infected with HMPV for 24 or 48 h, then detached, stained with CM-H2DCFDA, and analyzed by flow cytometry. (**a**) Overlay histograms show CM-H2DCFDA fluorescence for blank control (red), uninfected (blue), and HMPV-infected (orange) macrophages, with quantification of MFI at 24 and 48 h post-infection. For the bacterial killing assay, macrophages were infected with HMPV and, at 48 h post-infection, viable cells were recovered, seeded in 24-well plates, and challenged with *S. aureus* (MOI 40). After 2 h, extracellular bacteria were eliminated with gentamicin (100 µg/mL), cells were lysed and intracellular bacteria were quantified by plating serial dilutions. (*n* = 3). (**b**) Representative agar plates show *S. aureus* recovered from uninfected and HMPV-infected macrophages, with quantification shown as log10 CFU/mL (**left**) and bacterial yield relative to uninfected controls (**right**; uninfected = 100%). (*n* = 6). Data are presented as mean ± SEM from the indicated independent experiments. Statistical significance was determined by Student’s *t*-test. * *p* < 0.05; *** *p* < 0.001.

Because reactive oxygen species (ROS) represent a key antimicrobial effector mechanism involved in intracellular bacterial processing, we next evaluated whether HMPV replication affects ROS production in macrophages. Cells were infected with HMPV for 24 or 48 h, harvested, and incubated with CM-H_2_DCFDA, an ROS-sensitive fluorescent probe that becomes fluorescent following esterase cleavage and oxidation. ROS levels were quantified by flow cytometry. Our results show that at 24 h post-infection, ROS Mean Fluorescent Intensity (MFI) was comparable between uninfected (231 ± 5) and HMPV-infected macrophages (247.3 ± 5.1), indicating no significant difference. In contrast, at 48 h post-infection, infected macrophages exhibited significantly higher ROS levels (233.7 ± 2.3) compared with uninfected controls (196.3 ± 9.2) (Figure 3a). Collectively, these data indicate that HMPV infection does not impair macrophage ROS production and may instead enhance ROS generation at later stages of infection.

To further determine whether the observed phagocytic defect resulted from impaired bacterial uptake or defective intracellular processing, we performed a bacterial killing assay. This approach allows discrimination between defects at the level of bacterial internalization versus intracellular survival. If HMPV-infected macrophages were unable to effectively process or eliminate internalized bacteria, an increase in colony-forming units (CFU) counts would be expected due to enhanced intracellular bacterial survival. In contrast, if the primary defect occurred at the uptake stage, fewer bacteria would be internalized, leading to a decrease in CFU recovery.

For this assay, macrophages were infected with HMPV in 6-well plates, and incubated for 48 h. To control for potential virus-induced cell death, only viable macrophages were reseeded into 24-well plates prior to bacterial challenge. Cells were then challenged with bacteria for 2 h. To selectively quantify intracellular bacteria, cell cultures were treated with gentamicin (100 µg/mL), an antibiotic that does not penetrate eukaryotic cells and effectively eliminates extracellular bacteria. Following antibiotic treatment, macrophages were lysed to release internalized bacteria, which were quantified by CFU enumeration. As shown in Figure 3b, representative agar plates revealed that the plate from uninfected macrophages was densely populated with *S. aureus* colonies, whereas that from HMPV-infected macrophages showed markedly fewer colonies. Quantitative analysis corroborated these observations, with intracellular bacterial counts decreasing from 2.97 ± 0.03 log_10_ CFU/mL in uninfected macrophages to 2.56 ± 0.02 log_10_ CFU/mL in HMPV-infected cells (Figure 3b), corresponding to a ~60% reduced bacterial yield. Together, these results indicate that HMPV infection significantly reduces the number of bacteria internalized by macrophages, suggesting that HMPV primarily impairs bacterial uptake rather than enhancing intracellular killing.

### 3.4. HMPV Infection Reduces CD36 Expression in Macrophages

Macrophages recognize and clear bacteria through multiple pattern-recognition and scavenger receptors, including CD36, which contributes to the recognition and uptake of pathogens such as *S. aureus* [44,45,46,47,48,49]. When CD36 expression or function is impaired, macrophages exhibit a reduced capacity to internalize and kill invading microbes, leading to increased susceptibility to infection [46]. However, it remains unclear whether HMPV affects macrophage phagocytic function by altering CD36 expression, thereby compromising bacterial phagocytosis.

**Figure 4:**
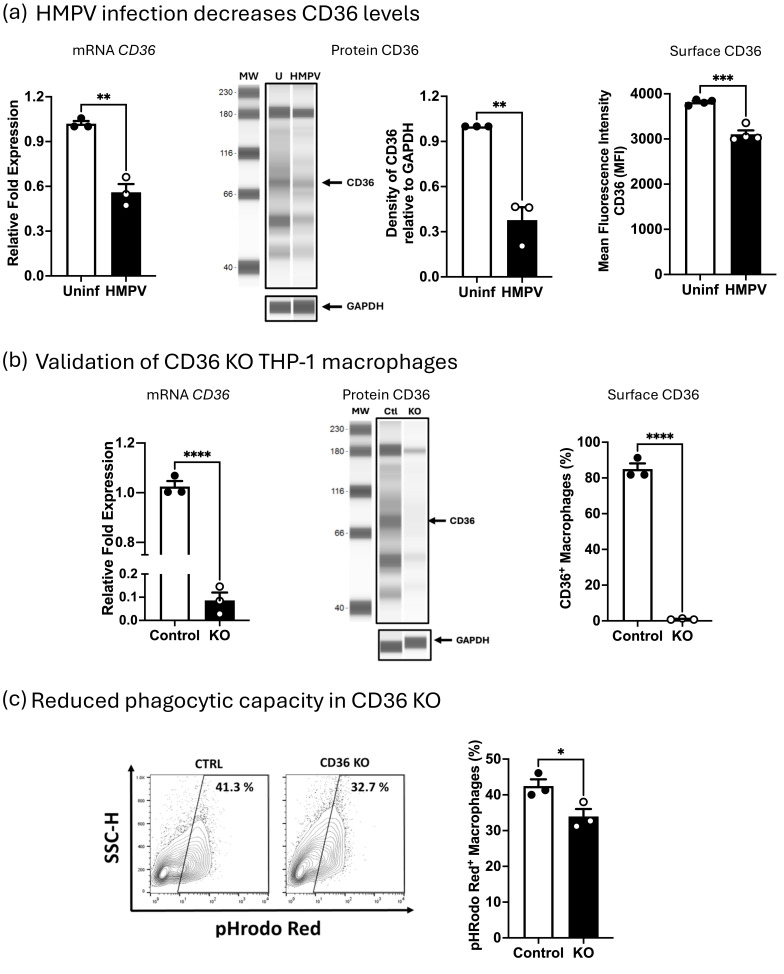
CD36 contribution in macrophage phagocytosis following HMPV infection. THP-1-derived macrophages were infected with HMPV and incubated for 48 h. (**a**) CD36 expression was evaluated at the mRNA, total protein, and cell surface levels. Transcript abundance was quantified by RT-qPCR (**left panel**), while total protein was measured by capillary Western blot with densitometric normalization to GAPDH (**middle**). Surface expression was determined by flow cytometry following anti-CD36 staining and reported as mean fluorescence intensity (MFI) (**right panel**). (**b**) CRISPR–Cas9 THP-1 CD36 knockout (KO) macrophages were validated by RT-qPCR analysis of CD36 mRNA expression (**left panel**), capillary Western blot analysis with densitometric normalization to GAPDH (**middle**), and flow cytometric quantification of CD36+ macrophages (**right panel**). (**c**) Parental THP-1 (Control) and CD36 KO macrophages were exposed to pHrodo-labeled *S. aureus* (MOI 40), and phagocytosis was quantified by flow cytometry. Representative phagocytosis dot plots (**left**) and corresponding quantification (**right**) show the percentage of pHrodo Red+ macrophages. Data are presented as mean ± SEM from the indicated independent experiments. Each dot represents an independent experiment (*n* = 3). U = uninfected; MW = molecular weight; Ctrl = Control; KO = knock out. Statistical significance was determined by Student’s *t*-test. * *p* < 0.05; ** *p* < 0.01; *** *p* < 0.001; **** *p* < 0.0001.

CD36 is a highly glycosylated scavenger receptor that plays a crucial role in the phagocytosis of *S. aureus* and other bacterial pathogens [42,44,45,46,47,48,50]. Therefore, to further test whether HMPV contributes to *S. aureus* entry via CD36, we characterized CD36 expression at the gene, protein, and functional surface levels in macrophages following HMPV infection. For gene expression analysis, total RNA was extracted from infected and uninfected macrophages at 48 h post-infection, and CD36 mRNA levels were quantified by RT-qPCR. Our results showed that HMPV infection reduced CD36 transcription from 1.0 ± 0.01 in uninfected cells to 0.56 ± 0.05-fold in infected macrophages, indicating a significant downregulation (Figure 4a, left panel). At the protein level, cell lysates from macrophages infected for 48 h were analyzed using capillary-based Western blotting. Consistent with the RT-qPCR results, densitometric analysis revealed a decrease in CD36 protein from the reference value of 1.0 in uninfected cells to 0.33 in HMPV-infected macrophages, confirming downregulation at the protein level (Figure 4a, middle panel). Since CD36 functions as a surface receptor, we next assessed its plasma membrane expression by flow cytometry. Macrophages were detached, Fc-blocked, and stained with an anti-CD36 antibody. HMPV-infected macrophages exhibited a decrease in MFI, indicating that the level of CD36 expression per cell are diminished (Figure 4a, right panel). Overall, these findings demonstrate that HMPV infection reduces CD36 expression at multiple levels, which may impair bacterial uptake and contribute to the observed phagocytic dysfunction in HMPV-infected macrophages.

To directly assess the role of CD36 in bacterial uptake, we generated a CD36 KO THP-1 cells using CRISPR-Cas9 gene editing. This approach allowed us to examine the impact of CD36 loss on bacterial phagocytosis. The KO was validated by confirming the absence of CD36 expression at both the gene and protein levels (Figure 4b). Following validation, we performed a phagocytosis assay to compare bacterial uptake between the parental THP-1 cells (Control) and CD36 KO macrophages. Macrophages were exposed to pHrodo-labeled bacteria for two hours, and bacterial uptake was quantified by flow cytometry. As observed in Figure 4c, in Control macrophages, 47.3% of cells were pHrodo-positive, whereas this percentage decreased to 32.7% in CD36 KO macrophages, representing a 14.6% reduction. While these findings demonstrate that CD36 contributes to bacterial phagocytosis in macrophages, the partial reduction in uptake indicates that alternative receptors participate in this process. Overall, CD36 deficiency partially reduced bacterial uptake, contributing to macrophage phagocytosis.

### 3.5. HMPV G Protein Mediates CD36 Downregulation and Reduced Phagocytosis

HMPV viral proteins are well recognized for their immunomodulatory functions, with surface glycoproteins in particular shaping host–pathogen interactions by altering immune recognition, signaling, or effector responses [51,52,53,54,55,56,57,58,59,60,61,62,63]. In HMPV, the non-lethal major proteins G, M2-2, and SH have been implicated in modulating host immune pathways [51,52,53,54,55,56,57,58,59,60,61,62,63]. Given that the reduction in phagocytosis we observed was replication-dependent, we reasoned that one or more of these proteins could actively contribute to CD36 downregulation and consequently impair bacterial uptake.

**Figure 5:**
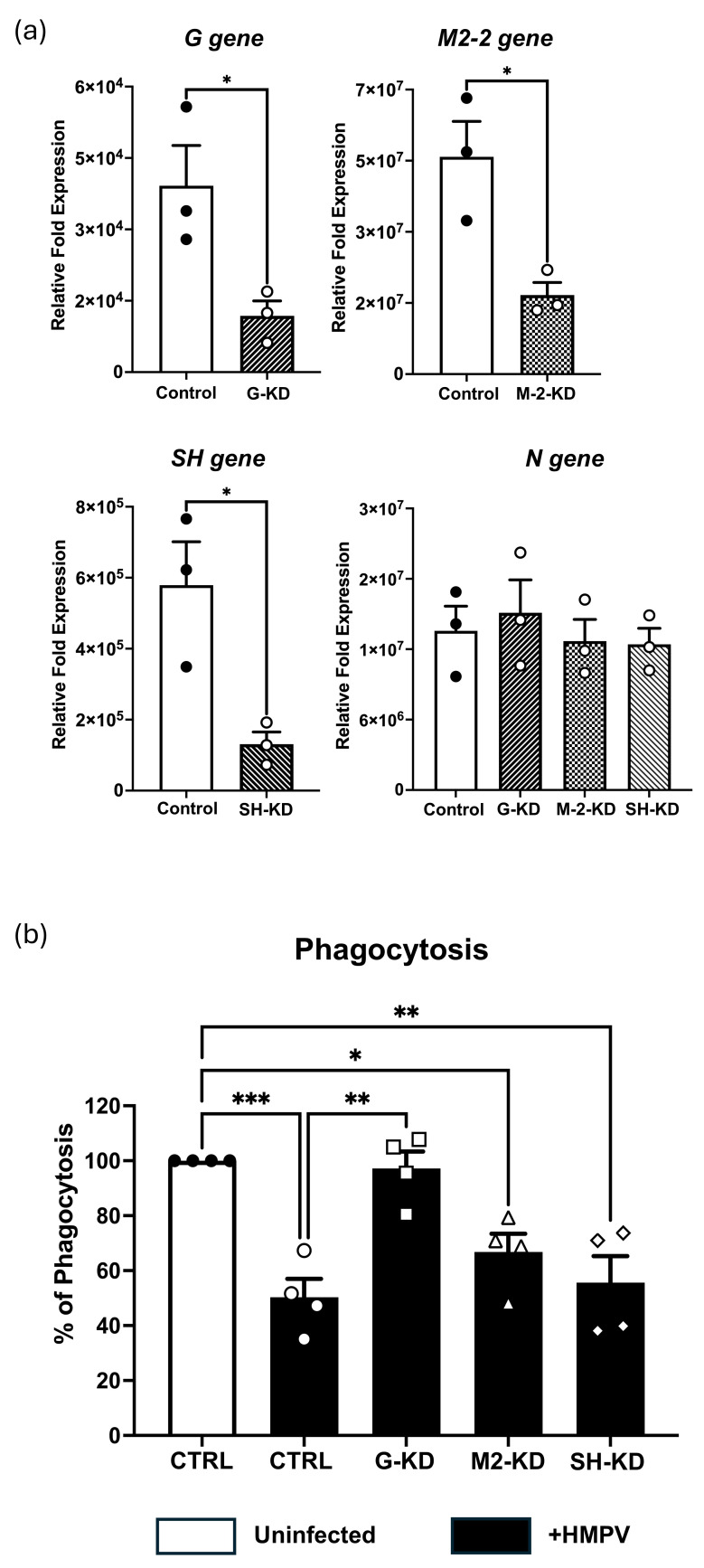
Effect of viral protein knockdown on macrophage phagocytosis. THP-1 cells stably expressing shRNA targeting the viral proteins G, M2-2, or SH were generated by lentiviral transduction, differentiated into macrophages, and infected with HMPV for 48 h. (**a**) Knockdown efficiency was confirmed by RT-qPCR analysis of viral gene expression of G, M2-2, SH. N gene expression was quantified as a marker of viral replication (*n* = 3). (**b**) Infected macrophages were exposed to pHrodo-labeled *S. aureus* (MOI 40) for 2 h, and phagocytosis was quantified by flow cytometry (*n* = 4). Relative phagocytosis was normalized to uninfected controls (100%). Data are presented as mean ± SEM from the indicated independent experiments. Statistical significance was determined by Student’s *t*-test or one-way ANOVA with Bonferroni multiple comparisons test * *p* < 0.05, ** *p* < 0.01, *** *p* < 0.001. KD = knockdown.

To test this, we generated THP-1 cell lines stably expressing short hairpin RNAs (shRNAs) targeting the viral G, M2-2, and SH, enabling specific knockdown (KD) of each protein. THP-1 parental cell line was used as control. To validate the KD cell lines, monocytes (THP-1 and KD) were differentiated into macrophages and infected with HMPV for 48 h. Total RNA was extracted, converted to cDNA, and analyzed by RT-qPCR for viral gene expression. As shown in Figure 5a, in HMPV-infected cells, G expression reached approximately 3.9 × 10^4^-fold compared with 1.2 × 10^4^-fold in G KD cells. M2-2 expression decreased from 5.2 × 10^7^-fold in THP-1 cells to 1.9 × 10^7^-fold in M2-2 KD cells, while SH expression decreased from 5.8 × 10^5^-fold to 1.3 × 10^5^-fold in SH KD cells. These results confirm efficient KD of the targeted viral genes. Notably, no significant differences were observed in the expression of the viral N gene among the different KD cell lines, indicating that overall viral replication was not substantially affected by the knockdown of G, M2-2, or SH.

To directly assess the impact of the G protein on bacterial phagocytosis, THP-1-derived macrophages were infected with HMPV at an MOI of 1.0 for 48 h. Cells were then detached; live cells were seeded and subsequently exposed to pHrodo-labeled bacteria at an MOI of 40 for 2 h. Phagocytosis was analyzed by flow cytometry and normalized to the uninfected control (set at 100%). Data shown in Figure 5b indicates that THP-1 HMPV-infected macrophages exhibited reduced phagocytosis (50.33 ± 6.7%), whereas G KD macrophages displayed nearly restored phagocytic activity (97.19 ± 6.15%). In contrast, M2-2 and SH KD macrophages showed reduced phagocytosis (67.58 ± 6.82% and 53.90 ± 10.72%, respectively). These findings suggest a role of the HMPV G protein in modulating phagocytic capacity.

### 3.6. HMPV Reduces Phagocytic Activity in MDMs

**Figure 6:**
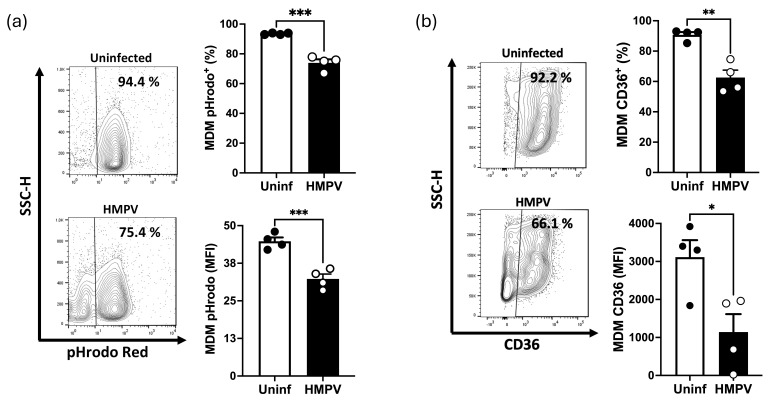
Phagocytosis of *S. aureus* by HMPV-infected MDMs. MDMs were infected with HMPV for 48 h. MDMs were then exposed to pHrodo-labeled *S. aureus* for 2 h, and phagocytosis was quantified by flow cytometry. (**a**) Phagocytosis assays were performed using viable macrophages. Representative contour plots (**left**) show the percentage of pHrodo^+^ macrophages in control (no *S. aureus*), uninfected, and HMPV-infected conditions. Corresponding quantification (**right**) shows the percentage of pHrodo^+^ MDMs. (**b**) CD36 expression was also quantified based on the expression of CD36 on macrophages. Representative contour plots are shown (**left**), with quantification of CD36 in MDM by percentage and MFI (**right**). Data are presented as mean ± SEM from the indicated independent experiments (*n* = 4). Statistical significance was determined by Student’s *t*-test. * *p* < 0.05, ** *p* < 0.01, *** *p* < 0.001.

To validate our findings in THP-1 cells, primary human MDMs were infected with HMPV at an MOI of 1.0. After 48 h of infection, macrophages were detached, counted, and re-seeded to control for potential cell death induced by infection and ensure that phagocytosis measurements were performed using viable macrophages. MDMs were challenged with pHrodo-labeled *S. aureus* for 2 h, and bacterial uptake was quantified by flow cytometry. The percentage of pHrodo^+^ macrophages was used as a measure of overall phagocytosis, whereas pHrodo MFI provided an estimate of the relative phagocytic signal intensity within the analyzed macrophage population. As shown in Figure 6a, representative contour plots demonstrated a reduction in the percentage of pHrodo^+^ macrophages following HMPV infection compared with uninfected cells. Quantitative analysis showed that phagocytosis decreased about 20% in HMPV-infected MDMs, confirming that HMPV infection compromises macrophage phagocytic function. The concomitant reduction in pHrodo MFI further supports a decrease in the magnitude of bacterial uptake signal after HMPV infection. Further analysis of the expression of CD36 by flow cytometry also indicated that HMPV reduced the percentage of MDMs expressing CD36 and the levels of CD36 expression (MFI) (Figure 6b).

### 3.7. HMPV Reduces the Expression of Scavenger Receptors in the Lungs of HMPV-Infected Mice

Because the lung is a highly complex tissue with multiple interacting cell types organized within a specialized alveolar architecture, macrophage behavior during viral infection is inherently dynamic and strongly influenced by the local tissue environment [64]. In this context, resident and recruited macrophage populations can fluctuate in abundance, activation state, and receptor expression, patterns that are difficult to recapitulate in simplified in vitro systems.

**Figure 7:**
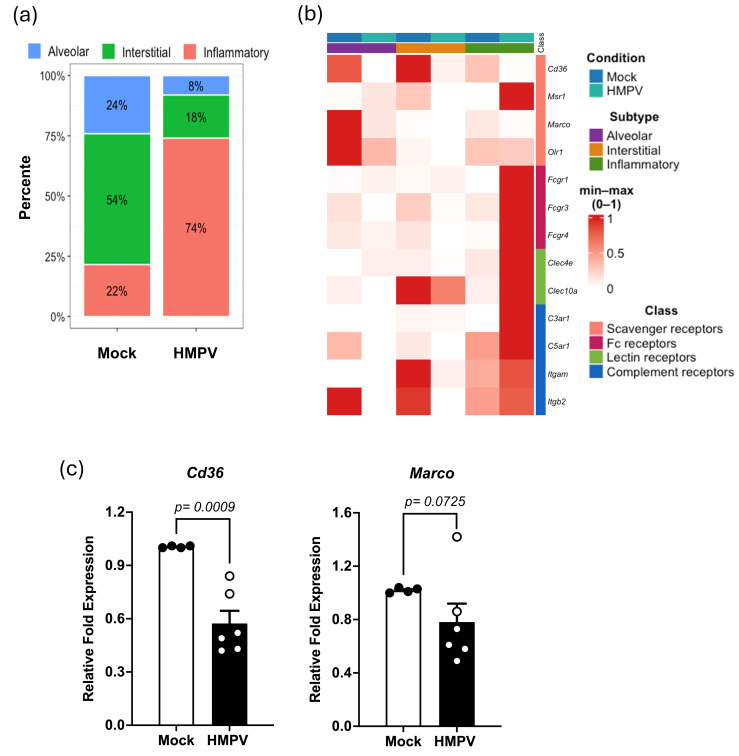
scRNA-seq analysis and RT-qPCR validation of phagocytosis-related receptors in HMPV-infected lungs. Publicly available scRNA-seq data from mock- and HMPV-infected mouse lungs were analyzed to assess macrophage subset distribution and phagocytosis-related gene expression. (**a**) Relative abundance of alveolar, interstitial, and inflammatory macrophage subsets in mock- and HMPV-infected lungs. (**b**) Heatmap showing scaled pseudobulk expression of selected phagocytosis-related receptors across macrophage subsets and conditions. (**c**) Independent validation of *Cd36* and *Marco* mRNA expression by RT-qPCR using total lung tissue from mock- and HMPV-infected mice. (**a**) Differential expression analyses were performed on pseudobulk counts generated by aggregating cells by biological sample within each macrophage subset prior to downstream normalization and comparative analyses (*n* = 2 biological replicates per condition). (**c**) Data are presented as mean ± SEM from 10 animals total, including 4 mock-infected and 6 HMPV-infected mice. Statistical significance was determined by Student’s *t*-test, with exact *p* values shown in the graphs.

As supporting evidence for our in vitro observations, we analyzed a publicly available scRNA-seq dataset generated from lungs of HMPV-infected mice at 1 day p.i. [40]. To assess the effects of HMPV infection on lung macrophage subsets, analyses were stratified into alveolar, interstitial, and inflammatory macrophage populations based on canonical marker expression, as described in the Methods. As shown in Figure 7a, interstitial macrophages represented approximately 54% of the macrophage compartment in mock lungs, whereas alveolar and inflammatory macrophages accounted for approximately 24% and 22%, respectively. Following HMPV infection, the macrophage landscape shifted toward a predominantly inflammatory phenotype, with inflammatory macrophages increasing to approximately 74%, while interstitial and alveolar macrophages decreased to 18% and 8%, respectively.

Heatmap analysis further revealed remodeling of macrophage phagocytic receptor programs following HMPV infection. Inflammatory macrophages displayed increased expression of Fc receptors (*Fcgr1*, *Fcgr3*, *Fcgr4*), lectin receptors (*Clec4e*, *Clec10a*), and complement-associated receptors (*C3ar1*, *C5ar1*), consistent with enhanced inflammatory and opsonic phagocytic pathways. In contratst, several scavenger receptors, including *Cd36*, *Olr1*, and *Marco*, were consistently reduced across alveolar, interstitial, and inflammatory macrophage populations in infected lungs relative to mock controls. Among these receptors, *Cd36* and *Marco* were prioritized for further validation based on their well-established roles in macrophage-mediated recognition and phagocytosis of *S. aureus* [46,65,66,67,68].

To validate the transcriptional changes observed in *Cd36* and *Marco*, total lung RNA was isolated from mock- and HMPV-infected mice 1 day p.i., and the expression of both scavenger receptors was quantified by RT-qPCR. Consistent with the transcriptomic analysis, *Cd36* expression was reduced in lungs from HMPV-infected mice, while *Marco* expression showed a similar downward trend compared with mock controls (Figure 7c), supporting HMPV-associated remodeling of macrophage scavenger receptor programs in the lung environment.

## 4. Discussion

Alveolar macrophages, the frontline sentinels in the lung, not only clear pathogens by phagocytosis but also shape downstream immunity. Clinical studies indicate that HMPV infection is associated with secondary bacterial pneumonia [23,69,70], yet the mechanisms by which the virus impairs macrophage antibacterial function remain unclear. Given the central role of phagocytosis, we assessed macrophage uptake after HMPV infection and observed a replication- and time-dependent defect: by 48 h post-infection, phagocytic capacity was reduced by ~60%. This demonstrates that HMPV actively impairs macrophage function, providing a plausible route for bacterial complications in vivo.

When we examined whether the reduction in phagocytosis during HMPV infection was mediated by extrinsic, cytokine-mediated effects or reflected a cell-intrinsic defect, we observed that transfer of UV-inactivated supernatants from HMPV-infected macrophages to uninfected cells did not impair phagocytosis, suggesting that secreted factors do not mediate this impairment. These findings contrast with reports in RSV and IAV infections, where IFN-β alone can reduce bacterial uptake, suggesting virus-specific differences in how IFN signaling impacts macrophage function [42]. To further assess the intrinsic effects of infection, we used HMPV-GFP to identify infected (GFP^+^) from uninfected (GFP^−^) macrophages within the same culture. Phagocytic activity was markedly reduced in GFP^+^ cells, demonstrating a cell-intrinsic defect. Similar induction-restricted impairments have been described for other viruses, including IAV and HIV-1, where active replication or expression of specific viral proteins is required to disrupt macrophage phagocytosis and receptor expression [42,71]. Together with the absence of phagocytic impairment upon exposure to UV-inactivated virus, our data indicate that reduced phagocytosis during HMPV infection depends on active replication and reflects direct, infection-dependent reprogramming of macrophage function rather than paracrine cytokine signaling alone.

Phagocytosis is a multistep, tightly regulated process encompassing pathogen recognition, internalization, and intracellular killing via phagolysosomal degradation, including, tightly regulated process encompassing pathogen recognition, internalization, and intracellular killing via phagolysosomal degradation, involving ROS and enzymatic mechanisms. Because our initial assays used pHrodo-labeled *S. aureus*, which fluoresces only in acidic phagolysosomes, it was unclear whether the reduced signal reflected impaired uptake or downstream processing. Analysis of ROS production revealed that early oxidative responses remain intact and, strikingly, late-stage ROS levels were elevated in infected cells. This argues against defective intracellular killing and suggests that early phagocytic steps, such as recognition or internalization, are primarily disrupted. Similar virus-induced ROS patterns have been observed with RSV [72] and IAV in epithelial cells [73], and with SARS-CoV-2 in primary murine and human macrophages. These findings [74] support the notion that intracellular antimicrobial mechanisms can remain functional despite impaired uptake.

Building on our observation that phagocytic impairment is strictly dependent on active HMPV replication and confined to infected cells, we next asked whether this defect occurred at the level of bacterial uptake or reflected an intracellular killing mechanism distinct from ROS. To address this, we performed a bacterial killing assay to quantify viable intracellular bacteria. Rather than observing increased bacterial survival, which would indicate defective intracellular processing, we detected a marked reduction in recoverable CFUs from HMPV-infected macrophages. This decrease suggests reduced bacterial internalization, as fewer bacteria enter infected cells in the first place. Together with the preserved or elevated ROS responses, these findings suggest that HMPV replication primarily disrupts early steps of phagocytosis, such as pathogen recognition or uptake, rather than downstream intracellular killing mechanisms.

Further analysis of CD36, a well-characterized scavenger receptor involved in the recognition and internalization of several bacteria [46,47,48,50] indicated that HMPV downregulates CD36 at the gene, protein and surface levels. To directly evaluate the role of CD36, we used a CD36 KO macrophage cell line and observed that CD36 contributes to *S. aureus* uptake. These findings demonstrate that CD36 contributes to bacterial phagocytosis in macrophages. However, the partial reduction in uptake suggests that additional receptors are also involved in this process, warranting further investigation. The current findings are consistent with studies on RSV and IAV, in which CD36 downregulation in macrophages correlates with impaired phagocytosis of Streptococcus pneumoniae [42]. However, in those studies, treatment with recombinant IFN-β was sufficient to reduce CD36 levels, suggesting a link between type I interferon signaling and the regulation of phagocytic receptors. However, in our model, UV-inactivated supernatants from HMPV-infected cells did not impair phagocytosis, suggesting that soluble factors alone cannot fully account for CD36 downregulation. One possible explanation for this discrepancy is that CD36 regulation may require sustained intracellular signaling events triggered by active viral replication or involve additional viral-mediated modulation of transcriptional or post-transcriptional mechanisms not mimicked by IFN treatment alone. This suggests that while type I IFN can contribute to CD36 repression, the full extent of downregulation likely involves virus-specific or replication-dependent mechanisms that go beyond cytokine signaling.

To further explore the mechanism by which HMPV impairs phagocytosis, we evaluated macrophages stably expressing shRNAs targeting individual HMPV genes (G, M2-2, and SH). RT-qPCR analysis confirmed efficient reduction in each targeted viral transcript, whereas N gene expression remained unchanged, indicating that shRNA expression did not affect viral replication in this macrophage model. These findings support a gene-specific effect of the shRNAs rather than a general reduction in infection. Notably, knockdown of G restored phagocytic capacity to levels comparable to those of uninfected controls, implicating the HMPV attachment protein as a potential mediator of impaired macrophage bacterial uptake. Nevertheless, because these experiments were performed in the context of viral infection, additional reductionist studies using G protein expression alone or alternative delivery approaches will be necessary to confirm its contribution and determine whether G protein is sufficient to impair phagocytosis independently of other viral factors.

Validation of the effect of HMPV on MDM phagocytosis and CD36 surface protein expression confirmed the downregulation effect induced by the viral infection. Although the reduction in phagocytosis was more moderate than that observed in THP-1-derived macrophages, this difference is consistent with published evidence showing that THP-1-derived macrophages and primary MDMs are not fully interchangeable models. PMA-differentiated THP-1 cells differ from MDMs in marker expression, cytokine secretion, surface activation phenotype, and phagocytic responses, and MDMs have been reported to display greater basal phagocytic capacity than THP-1 cells [75]. Moreover, primary human macrophages differentiated with GM-CSF or M-CSF exhibit distinct phenotypes, viability, and pathogen-specific phagocytic activity, further indicating that macrophage origin and differentiation state shape functional responses to infection [76]. Thus, the reduction observed in MDMs supports the notion that HMPV impairs bacterial phagocytosis.

We next examined whether these molecular changes extend to heterogeneous macrophage populations in the lung. scRNAseq analysis of infected mice revealed profound remodeling of alveolar, interstitial, and inflammatory macrophages during HMPV infection. Resident alveolar and interstitial macrophages were reduced, while inflammatory macrophages expanded, consistent with recruitment and differentiation of monocyte-derived cells during antiviral inflammation in the lung [77,78,79,80]. This shift is expected to affect overall phagocytic capacity, given the specialized homeostatic and scavenging functions of resident macrophages. Furthermore, we found that HMPV infection also induced subtype-specific reprogramming of phagocytic receptors. Heatmap analysis of curated receptor families showed a broad suppression of scavenger receptors, including Cd36, Marco, and Olr1, whereas several Fc and lectin receptors were selectively upregulated in inflammatory macrophages. Based on these findings, we selected *Cd36* and *Marco* for validation because they were among the scavenger receptors most consistently reduced across alveolar, interstitial, and inflammatory macrophage populations and because both have established roles in macrophage recognition and uptake of bacteria, including *S. aureus* [46,65,66,67,68]. RT-qPCR analysis was then performed using RNA extracted from whole lung tissue, which represents a mixed population of resident and recruited immune and structural cells. Despite this cellular complexity, *Cd36* expression was significantly reduced, and *Marco* showed a trend toward decreased expression in HMPV-infected lungs, consistent with the reduced scavenger receptor expression observed across macrophage populations in the scRNA-seq analysis. These findings further support the notion that HMPV infection disrupts scavenger receptor pathways involved in bacterial recognition.

In summary, we have shown that HMPV actively impairs macrophage phagocytosis through a replication-dependent, cell-intrinsic mechanism, with evidence suggesting that the viral G protein contributes to this effect. This disruption occurs primarily at the level of bacterial uptake rather than intracellular killing and is associated with reduced CD36 expression, which may contribute to the observed impairment in phagocytic function. Together, these findings provide a potential mechanistic explanation for the heightened susceptibility to secondary bacterial infections during HMPV infection and underscore the importance of virus-induced modulation of macrophage receptors and phagocytic pathways in viral pathogenesis.

## 5. Conclusions

HMPV compromises macrophage-mediated bacterial uptake through a replication-dependent mechanism associated with altered expression of phagocytic receptors, providing a potential explanation for increased susceptibility to secondary bacterial infections.

## Data Availability

The data supporting the conclusions of this research manuscript are all present within the article.
